# Knocking Out Chloroplastic Aldolases/Rubisco Lysine Methyltransferase Enhances Biomass Accumulation in *Nannochloropsis oceanica* under High-Light Stress

**DOI:** 10.3390/ijms25073756

**Published:** 2024-03-28

**Authors:** Wensi Liang, Li Wei, Qintao Wang, Wuxin You, Ansgar Poetsch, Xuefeng Du, Nana Lv, Jian Xu

**Affiliations:** 1 Single-Cell Center, CAS Key Laboratory of Biofuels, Shandong Key Laboratory of Energy Genetics, Qingdao Institute of BioEnergy and Bioprocess Technology, Chinese Academy of Sciences, Qingdao 266101, China; liangws@qibebt.ac.cn (W.L.);; 2 University of Chinese Academy of Sciences, Beijing 100049, China

**Keywords:** lysine methyltransferase, CRISPR/Cas9, carbon fixation, Calvin cycle, *Nannochloropsis* spp.

## Abstract

Rubisco large-subunit methyltransferase (LSMT), a SET-domain protein lysine methyltransferase, catalyzes the formation of trimethyl-lysine in the large subunit of Rubisco or in fructose-1,6-bisphosphate aldolases (FBAs). Rubisco and FBAs are both vital proteins involved in CO_2_ fixation in chloroplasts; however, the physiological effect of their trimethylation remains unknown. In *Nannochloropsis oceanica*, a homolog of LSMT (NoLSMT) is found. Phylogenetic analysis indicates that NoLSMT and other algae LSMTs are clustered in a basal position, suggesting that algal species are the origin of LSMT. As NoLSMT lacks the His-Ala/ProTrp triad, it is predicted to have FBAs as its substrate instead of Rubisco. The 18–20% reduced abundance of FBA methylation in NoLSMT-defective mutants further confirms this observation. Moreover, this gene (*nolsmt*) can be induced by low-CO_2_ conditions. Intriguingly, NoLSMT-knockout *N. oceanica* mutants exhibit a 9.7–13.8% increase in dry weight and enhanced growth, which is attributed to the alleviation of photoinhibition under high-light stress. This suggests that the elimination of FBA trimethylation facilitates carbon fixation under high-light stress conditions. These findings have implications in engineering carbon fixation to improve microalgae biomass production.

## 1. Introduction

Microalgae, renowned for their ability to efficiently convert solar energy and CO_2_ into biomass, are recognized as a promising and sustainable source of energy and a diverse array of high-value products. They have demonstrated utility across multiple sectors, including energy, pharmaceuticals, industrial chemicals, and nutraceuticals [1,2]. In particular, due to the continuous increase in atmospheric CO_2_ levels caused by human activities, it is imperative to explore and develop strategies for enhancing the carbon fixation potential of microalgae [3]. However, the limited availability of effective genetic modification targets leads to relatively slow improvement in the carbon fixation efficiency of microalgae.

The Calvin cycle is the principal pathway for carbon fixation, converting CO_2_ into organic compounds [4]. Modifying key enzymes in this cycle through genetic engineering effectively improves carbon fixation efficiency [5,6,7]. The presence of co- and post-translational modifications is a significant concern in the engineering of more efficient enzymes in the Calvin cycle [8,9,10]. Seven chloroplastic proteins functioning in CO_2_ assimilation are found to be methylated in *Arabidopsis* [11]. One of the enzymes responsible for epigenetic modification is Rubisco large-subunit methyltransferase (LSMT), which is located in chloroplasts and trimethylates Lys14 of Ribulose 1,5-bisphosphate carboxylase/oxygenase (Rubisco) or chloroplastic fructose-1,6-bisphosphate aldolase (FBA), two key proteins involved in the Calvin cycle that control carbon flux [10,12,13]. Therefore, LSMT presumably regulates carbon fixation efficiency by altering the methylation form of Rubisco or FBA.

The LSMT protein is a member of SET domain containing methyltransferases [9,14,15]. The ancestral function of LSMT is FBA trimethylation. In a recent event in the course of higher plant evolution, this function emerged in the ancestors of Fabaceae, Cucurbitaceae, and Rosaceae to include Rubisco large subunit (RBCL) as an additional substrate to the archetypal enzyme. The central motif in LSMT enzymes, known as the His-Ala/ProTrp triad, confers the ability to trimethylate Rubisco [16]. Although LSMT has the potential to regulate carbon fixation, its function still requires further verification. *Arabidopsis thaliana lsmt*-knockout mutants are viable and do not present obvious macroscopic phenotypes when grown under standard conditions. Moreover, trimethylation does not modify the catalytic properties and oligomeric states of chloroplastic FBAs in vitro [17]. Knock-down mutants of tobacco plants with decreased expression of the bifunctional LSMT gene exhibit no alteration in the growth phenotype, and show no difference in CO_2_ assimilation rates when compared to wild-type plants [18]. Therefore, it is crucial to obtain gene-deficient mutants and identify culture conditions that can induce phenotypic differences between wild-type and mutant strains in order to investigate the function of LSMT.

*Nannochloropsis* spp. are unicellular, photosynthetic microalgae belonging to the heterokont group [19]. The ability of *Nannochloropsis* spp. to produce significant quantities of triacylglycerides and high-value products, including eicosapentaenoic acid, under various environmental conditions and at large scales, has attracted industrial interest [20]. Exceptional genetic tools, such as random insertional mutagenesis [21], gene overexpression [22], homologous recombination [23], RNAi-based gene knockdown [24], and Cas9-based gene knockout [25], have greatly facilitated mechanistic investigations for transcriptional regulation [26], the carbon concentrating mechanism [27], photosynthesis [28], phytohormone function [29], carbon partitioning [30], oil metabolism [31], etc. Therefore, *Nannochloropsis* spp. are an excellent group of model algae for functional gene research and genetic engineering for feedstock development.

Here, a homologous protein of LSMT in *Nannochloropsis oceanica* (NoLSMT) was induced in transcription by low-CO_2_ conditions, implying a possible role in carbon fixation. Phylogenetic analysis indicates that LSMT originated from algae species. Sequences alignment and Western blot detection suggest that FBAs rather than Rubisco are the substrate of NoLSMT in *Nannochloropsis oceanica.* We used a CRISPR/Cas9-mediated method to knock out NoLSMT to characterize its function. Intriguingly, we observed significantly improved biomass accumulation and the alleviation of photoinhibition in knockout mutants under the high-light condition, suggesting that the removal of FBA trimethylation presumably facilitates carbon fixation in high-light stress to avoid photodamage. These findings provide insight into the functional role of protein methylation modifications in the Calvin cycle, as well as their potential contribution to engineering carbon fixation for enhanced microalgae biomass production.

## 2. Results

### 2.1. NoLSMT Contains the Key Enzymatic Domains to Trimethylate Rubisco or FBAs in Chloroplasts

**Figure 1:**
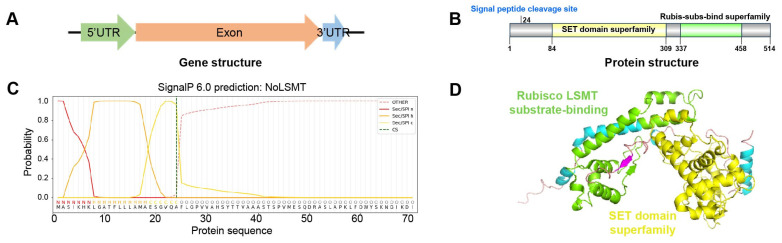
Sequence analysis of NoLSMT. (**A**) Gene structure of *nolsmt*. The green, red, and blue arrows represent 5′ untranslated region (5′UTR), exon region, and 3′ untranslated region (3′UTR). (**B**) The protein structure of NoLSMT. The yellow and green regions represent the SET domain superfamily and the Rubis-subs-bind superfamily. (**C**) SignalP was used to predict the signal peptide cleavage sites of NoLSMT. The green dashed line represents the signal peptide cleavage site in NoLSMT: between the 24th and 25th amino acids. (**D**) The model of NoLSMT was built using the structure of Rubisco large-subunit methyltransferase, substrate-binding domain protein from *Nannochloropsis gaditana* as a template (Swiss model). The value of global model quality estimation (GMQE) is 0.83. The Rubisco LSMT substrate-binding domain is marked in green and SET domain is marked in yellow.

From the *N. oceanica* strain IMET1 genome, a predicted nuclear-encoded LSMT (NoLSMT) was identified, which shares 31.7% identity in its amino acid sequence with AtLSMT, a protein characterized as LSMT in *Arabidopsis thaliana* (Figure 1A,B). The sequencing of cDNA derived from total RNA reveals a 514-aa, ~56-kDa protein that is predicted to target chloroplasts (with a probability value of 0.2703 from HECTAR) and whose signal peptide cleavage site is predicted to be between the 24th and 25th aa (with a probability value of 0.9218 from SignalP) (Figure 1C). The predicted topology of NoLSMT reveals the presence of two functionally important domains: the SET domain superfamily (84–309 aa) and Rubisco LSMT substrate-binding domain (337–458 aa) (Figure 1D). 

### 2.2. Sequence Alignment and Phylogenetic Analysis Predict FBAs Rather Than Rubisco to Be Trimethylated Substrates of NoLSMT

**Figure 2:**
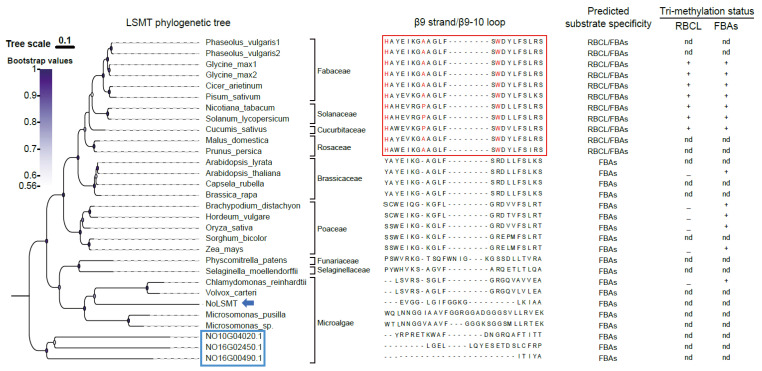
Phylogenetic analysis predicts FBAs to be substrates for trimethylation by NoLSMT. The sequence of the β9 strand and β9-β10 loop, inferred by sequence alignment and LSMT structure modeling using Protein Homology/AnalogY Recognition Engine V2.0 through the Phyre^2^ server, is presented alongside the organism name (www.sbg.bio.ic.ac.uk/phyre2/html/page.cgi?id=index, accessed on 11 March 2024) [32]. Sequences with the triad motif His-Ala/Pro-Trp are boxed in red. The red letters H, A, P, and W in the red box respectively stand for the abbreviations His, Ala, Pro, and Try. The predicted substrate specificity of LSMT enzymes correlates well with the available data on the trimethylation status of RBCL and FBAs in the corresponding organisms [12,17]. +, trimethylated; –, unmethylated; nd, not determined. NoLSMT is marked with blue arrows. The three other proteins containing the SET domain and Rubisco LSMT substrate-binding domain are boxed in blue. See Table S2 for the list of proteins obtained from the Uniport and Phytozome databases.

The triad motif His-Ala/Pro-Trp is regarded to be a characteristic feature of LSMT’s RBCL-methylating activity, and is located at the base of the β9 strand and β9–10 loop [16]. To predict the trimethylated substrate of NoLSMT, we performed sequence alignment and phylogenetic analysis. NoLSMT and other algae LSMTs are clustered together in a very basal position, indicating that LSMT originates from microalgae species. Loss of the triad motif His-Ala/Pro-Trp suggests that the FBAs rather than Rubisco are the trimethylated substrate of LSMTs from microalgae, including NoLSMT (Figure 2). In addition to NoLSMT, the *N. oceanica* genome also encodes three other proteins that contain the SET domain and Rubisco LSMT substrate-binding domain. Phylogenetic analysis suggests that these proteins diverged from the ancestral sequence early in evolutionary history, implying that their sequence and function are more conserved across different species (Figure 2). These paralogous proteins may also play a role in methylating FBAs or Rubisco, potentially regulating carbon fixation.

### 2.3. Two NoLSMT Knockout Lines Are Obtained by a CRISPR/Cas9-Mediated Method

**Figure 3:**
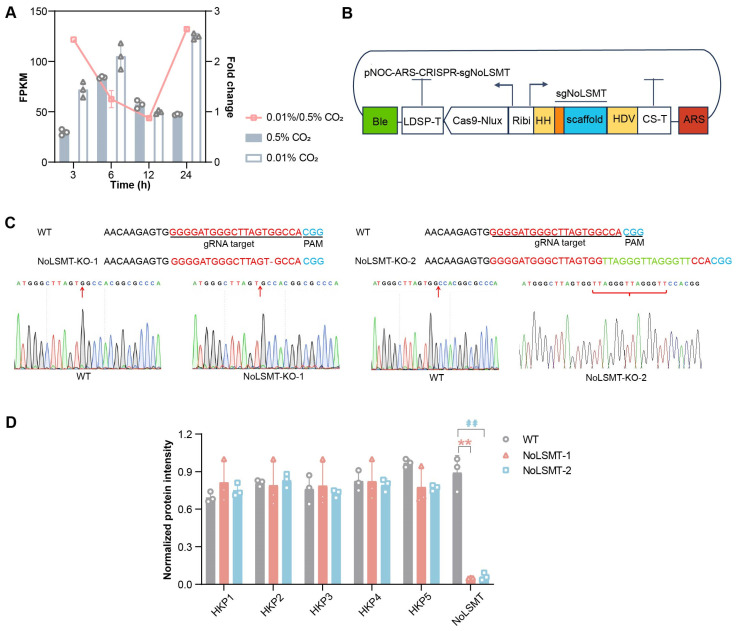
NoLSMT is knocked out by genome editing using Cas9/gRNA. (**A**) Abundance of NoLSMT transcripts under 0.01% and 5% CO_2_. Fold change is calculated as (0.01%-CO_2_)/(5%-CO_2_). FPKM: fragments per kilobase of exon model per million mapped reads. The triangle and circle represent the FPKM values of three technical replicates under 5% and 0.01% CO_2_ conditions respectively. (**B**) The *S. cerevisiae* CEN/ARS6 region (ARS, red) is included in the pNOC-ARS-CRISPR construct for episomal maintenance. Guide sequences (orange) for NoLSMT targets, with a 5′hammerhead ribozyme (HH), are fused to the gRNA scaffold to form the NoLSMT sgRNAs (sgNoLSMT). Ribozymes are highlighted in yellow. sgNoLSMTs are added to pNOC-ARS-CRISPR to form pNOC-ARSCRISPR-sgNoLSMT. *N. oceanica* is transformed with circular episomal CRISPR constructs. (**C**) Genotypic validation of mutants. Single-base-deletion mutagenesis: NoLSMT-KO-1; fourteen-base-insertion mutagenesis: NoLSMT-KO-2. The red letters represent the target guide RNA (gRNA) sequence, the blue letters represent the Protospacer Adjacent Motif (PAM) sequence, the red vertical line represents the deleted base, and the green letters represent the inserted bases. Red arrows indicate sites of base insertion and deletion, while red curly braces indicate the inserted bases. See Table S1 for primer sequences used for plasmid construction and mutant screening. (**D**) Normalized intensity of “housekeeper protein” (HKP) and target protein (NoLSMT). The expression intensity of proteins is normalized by peak area/maximum value of each protein. See Table S3 for target polypeptide sequences of NoLSMT and “housekeeper proteins”. Significant differences between WT and NoLSMT-1 are denoted by asterisks, and significant differences between WT and NoLSMT-2 are denoted by hashtags. Double asterisks (**) and double hashtags (##) indicate *p* values < 0.01.

To investigate the potential role of NoLSMT in carbon fixation, *N. oceanica* cells were first cultured under 5% CO_2_, and then, two equal aliquots were separately inoculated into 0.01% and 5% CO_2_ culture, respectively (Section 4). The transcript abundance of NoLSMT was analyzed (based on mRNA-Seq) 3, 6, 12, and 24 h after inoculation into 0.01% CO_2_ culture, and then, compared with those at the corresponding time points in the paralleled 5% CO_2_ culture. Under 0.01% CO_2_, the *nolsmt* transcript exhibited upregulation at 3 h, 6 h, and 24 h, peaked at 24 h (2.64-fold), and showed slight downregulation at 12 h (12.6%) (Figure 3A). Theis result shows that the transcript abundance of *nolsmt* is induced by low carbon, indicating that NoLSMT may play a role in carbon fixation.

To investigate the in vivo function of NoLSMT, we used a CRISPR/Cas9-mediated method to knock it out (Figure 3B). Two knockout mutants were obtained, which were validated at both the DNA and protein levels. DNA sequencing suggests that one mutant is due to single-base-deletion mutagenesis (NoLSMT-KO-1) and the other due to the insertion mutagenesis of fourteen bases (NoLSMT-KO-2) (Figure 3C). Moreover, to confirm successful NoLSMT knockout in vivo, total proteins from the two mutants and WT were extracted and digested in gel. We identified five proteins that are stably and abundantly expressed under various conditions and refer to them as “housekeeper proteins”. The expression levels and retention time of these “housekeeper proteins” and the target protein (NoLSMT) were measured using UPLC + TSQ (Triple Quadrupole Mass Spectrometry) and analyzed with Skyline 6.6.0 (https://skyline.ms/project/home/software/Skyline/begin.view). Only in the WT are unique peptides of NoLSMT detected (Figure 3D). In the mutant samples, peptides are not found. On the other hand, the expression of “housekeeper proteins”, which served as the negative control, are similar between the WT and mutants. Therefore, the NoLSMT protein was successfully knocked out in the mutants.

### 2.4. The Loss of FBA Trimethylation Due to NoLSMT Knockout Elevates Carbon Fixation Rate in High-Light Stress 

**Figure 4:**
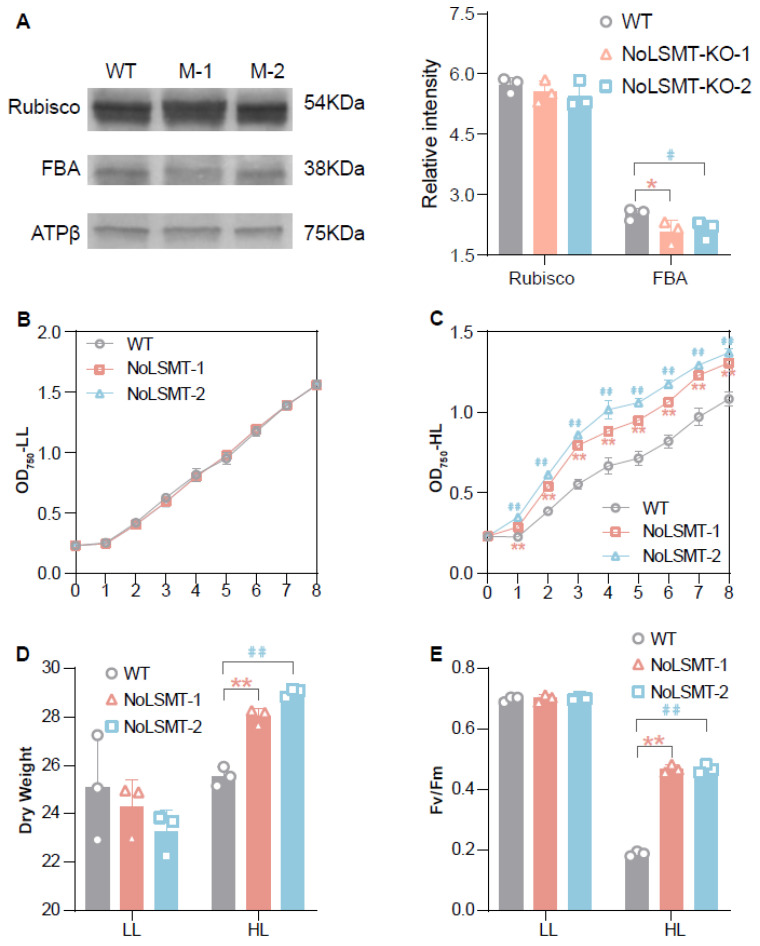
NoLSMT knockout lines exhibit enhanced high-light tolerance. (**A**) Immunodetection of proteins cross-reacting with polyclonal antibodies specific to N-terminal methylated lysine. Band intensities for individual proteins were normalized with that of ATPβ (ImageJ 1,53e; https://imagej.net/ij/). Values shown are the mean ± standard deviation (SD) from three technical replicates. (**B**) Growth curves under low-light condition. (**C**) Growth curves under high-light condition. (**D**) Dry weight after cultivation for six days under low-light and high light-condition. (**E**) The ratio of variable fluorescence to maximal fluorescence, which reflects the optimal/maximal photochemical efficiency of Photosystem Ⅱ (Fv/Fm). Values shown are the mean ± standard deviation (SD) from three biological replicates. Significant difference between mutants and WT is marked with asterisks or hashes (with ‘*’ or ‘#’ indicating *p* values < 0.05, and ‘**’ or ‘##’ indicating *p* values < 0.01).

To investigate the methylation status of Rubisco and FBAs in NoLSMT-defective mutants, Western blot analyses were performed. A commercial antibody specific to N-terminal methylated lysine was used to probe soluble extracts from cells. This antibody proves to be efficient to rapidly evaluate the methylation status of abundant proteins such as Rubisco and chloroplastic FBAs in *A. thaliana*, spinach, and pea chloroplast extracts (molecular mass of RBCL and chloroplastic FBA isoforms were identified in these species) [16]. Methylation is still detected at the position of FBAs in NoLSMT-defective mutants (Figure 4A). However, the methylation abundance of mutants decreases by 18–21% compared to the WT, indicating that FBAs are the substrates involved in methylation by NoLSMT. The three other proteins may also contribute to the methylation of FBAs in *N. oceanica*. Therefore, methylation modification remains in the NoLSMT-defective mutants, with a slightly lower level of methylation compared to the WT. Furthermore, RBCL is methylated in both the WT and mutants with no difference in abundance, suggesting that RBCL is not a substrate involved in methylation by NoLSMT. It is possible that RBCL is methylated by the three other proteins that contain the SET domain and Rubisco LSMT substrate-binding domain (Figure 4A).

To characterize the impact of losing NoLSMT on biomass accumulation, we measured the growth rate and dry weight of the knockout mutants under low light (LL; 20 µmol photons m^−2^ s^−1^). However, no apparent differences are detected, implying that the loss of FBA trimethylation has no effect on carbon fixation under LL (Figure 4B). To determine whether photosynthetic activity is inhibited by NoLSMT knockout, the maximum efficiency of Photosystem Ⅱ (Fv/Fm) under low light (LL) and high light (HL; 1100 µmol photons m^−2^ s^−1^) was evaluated using Imaging-PAM. Intriguingly, a significant increase (*p* < 0.01) in Fv/Fm is observed in the mutants after 48 h of high-light stress (Figure 4E), exhibiting enhanced HL tolerance due to the loss of FBA trimethylation. In addition, compared with the WT, the growth rates (represented by values of OD_750_) of the mutants increase significantly and the dry weight of the mutants increases by 9.7–13.8% (Figure 4C,D), indicating an increase in biomass accumulation under HL conditions. Therefore, the loss of FBA trimethylation due to NoLSMT knockout improves the carbon fixation rate in HL stress, and thus, alleviates photoinhibition and photodamage caused by excess excitation energy. Altogether, these results suggest NoLSMT induces the trimethylation of FBAs and exerts a negative regulatory effect on growth under HL stress.

## 3. Discussion

In this study, we characterized the function of NoLSMT in *N. oceanica*. Our phylogenetic analysis reveals that LSMT originates from algae species. Sequences alignment and Western blot detection suggest that FBAs rather than Rubisco are the trimethylated substrate of NoLSMT in *Nannochloropsis*. Furthermore, we discovered that low-CO_2_ conditions induced the expression of NoLSMT, suggesting a potential role in carbon fixation. By using CRISPR/Cas9-mediated gene knockout, we obtained NoLSMT knockout mutants and observed improved biomass accumulation and the alleviation of photoinhibition under high-light conditions, indicating that the loss of FBA trimethylation facilitates carbon fixation in high-light stress.

NoLSMT methylates the enzymes RuBisCo or FBAs with trimethylation modifications, which are key enzymes in the Calvin cycle. This interaction potentially regulates CO_2_ fixation by modifying the enzymes, enabling adaptation to diverse environments. However, disrupting or reducing the expression of LSMT does not impact plant growth in *A. thaliana* and tobacco plants when grown under standard conditions [17,18]. In *N. oceanica*, we observed upregulated expression of NoLSMT under low-CO_2_ conditions. It is possible that the NoLSMT-mediated trimethylation of FBA regulates its activity or interactions with other enzymes in the Calvin cycle, thereby influencing carbon fixation efficiency in *N. oceanica*. 

Notably, the knockout mutants of NoLSMT exhibit improved biomass accumulation and enhanced tolerance to high-light stress. This suggests that the loss of FBA trimethylation increases the efficiency of carbon fixation and reduces photoinhibition and photodamage caused by excess excitation energy. The exact mechanism underlying this phenomenon is still unclear and requires further investigation. It is possible that FBA trimethylation negatively regulates the activity or stability of FBAs, and its removal leads to increased carbon fixation capacity. Alternatively, FBA trimethylation may affect the interaction of FBAs with other proteins involved in the Calvin cycle, thereby influencing carbon flux and photosynthetic efficiency. Further studies are required to elucidate the molecular mechanisms underlying the observed phenotypes in the knockout mutants.

Methods for enhancing microalgal biomass production include optimizing growth conditions such as light intensity, temperature, and nutrient availability [33]. Additionally, strategies to improve carbon fixation efficiency have focused on the genetic engineering of key enzymes involved in the Calvin cycle, such as Rubisco [34,35]. However, our study highlights a novel approach of targeting the trimethylation of FBAs through NoLSMT manipulation, which has shown promising results in enhancing biomass accumulation and stress tolerance in *Nannochloropsis oceanica* (Table 1). In recent years, advances in synthetic biology have provided new tools and opportunities for engineering microalgal metabolism to improve carbon capture and utilization efficiency [36]. For instance, metabolic engineering strategies involving the manipulation of carbon fixation pathways, such as the introduction of high-efficiency carbon concentrating mechanisms, have shown potential for enhancing biomass production in microalgae [37,38]. Integrating the findings from our study on the regulatory role of NoLSMT in carbon fixation with these cutting-edge metabolic engineering approaches could further boost the development of sustainable and efficient microalgal biofuel production systems.

One promising application of the insights obtained from investigating NoLSMT in *Nannochloropsis oceanica* is the development of targeted biotechnological approaches to enhance carbon capture in microalgae-based carbon sequestration systems. By harnessing the knowledge of how NoLSMT impacts carbon fixation efficiency and biomass accumulation, researchers can potentially engineer microalgae strains with improved abilities to capture and store atmospheric CO_2_. For instance, through genetic modifications such as the overexpression or knockout of NoLSMT, researchers can manipulate FBA trimethylation to boost carbon fixation rates and enhance biomass productivity across various environmental conditions. These engineered microalgae could be deployed in bioremediation initiatives to reduce greenhouse gas emissions by sequestering CO_2_ from industrial sources or the atmosphere. This application illustrates how foundational research on the regulatory function of NoLSMT in carbon fixation pathways may offer tangible benefits in combating climate change and advancing sustainable biofuel production through refined carbon capture and utilization strategies.

## 4. Materials and Methods

### 4.1. Cell Strains and Growth Conditions

*Nannochloropsis oceanica* IMET1 was introduced into a customized f/2 liquid medium composition comprising 30 g/L sea salt and 10 mM Tris-HCl adjusted to a pH of 7.6. Additionally, the medium included 427.5 mg/L NaNO_3_, 30 mg/L NaH_2_PO_4_·H_2_O, a trace metal mixture (composed of 4.36 g/L Na_2_EDTA·2H_2_O, 3.15 g/L FeCl_3_·6H_2_O, 10 mg/L CoCl_2_·6H_2_O, 22 mg/L ZnSO_4_·7H_2_O, 180 mg/L MnCl_2_·4H_2_O, 9.8 mg/L CuSO_4_·5H_2_O, and 6.3 mg/L Na_2_MoO_4_·2H_2_O), and a vitamin stock solution (comprising 1 mg/L vitamin B12, 1 mg/L biotin, and 200 mg/L thiamine). The above reagents were all purchased from Sigma-Aldrich (St. Louis, MO, USA). For growth phenotype detection including the growth curve, dry weight, and chlorophyll fluorescence parameters, cell inoculation (initial concentration: 2.6 × 10^7^ cells/mL) was carried out in a 250 mL conical flask for agitation culture at 200 rpm and 25 °C under either high-light (HL, around 1000 µmol photons m^−2^ s^−1^) or low-light (LL, approximately 15 µmol photons m^−2^ s^−1^) conditions (ZQZY-CS8V, Shanghai Zhichu Shaker, Shanghai, China). Dry cell weight was determined using the standard filter paper method [34]. Briefly, 20 mL of algal solution was filtered through pre-dried filter paper (1440-055 Grade 40, Whatman, Maidstone, UK), dried at 105 °C for at least 10 h, and weighed after drying, and we subtracted the weight of the filter paper to obtain the dry weight of the 20 mL algal solution. 

### 4.2. Phylogenetic Analysis of LSMT Collected from the Uniport and Phytozome Databases

Phylogenetic analysis of NoLSMT was conducted utilizing amino acid sequences retrieved from the Uniport and Phytozome databases, employing standalone BLAST (with an E-value cutoff of 1 × 10^−6^) [48]. The selected sequences are detailed in Table S2. Alignment of the sequences was achieved using MAFFT with default parameters [49]. Subsequently, trimAl (-automated1) was applied to eliminate gaps and ambiguously aligned sites [50]. PhyML3 [51] (-d aa -b 1000 -m LG + G+F -f m -v e -a e) and ProtTest3 [52] (-all-distributions -F -AIC -BIC -tc 0.5) were used for phylogenetic analyses. Bootstrap support values were estimated using 1000 pseudo-replicates.

### 4.3. Experiment Comparing nolsmt Expression between 0.01% and 5% CO_2_ Culture Conditions 

*Nannochloropsis oceanica* IMET1 cells were cultivated in liquid cultures exposed to continuous light (approximately 80 ± 5 µmol photons m^−2^ s^−1^) at 25 °C and aerated with 5% CO_2_. Upon reaching the mid-logarithmic growth phase (OD_750_ of 2.6), cells were harvested, washed thrice with sterile seawater, and then, re-inoculated into fresh liquid medium at the same OD_750_ under light intensity of 80 ± 5 µmol photons m^−2^ s^−1^. Six replicate column reactors were employed for pre-adaptation under 5% CO_2_ conditions. Following a one-hour incubation period, three replicates were maintained under 5% CO_2_ while the other three replicates were transitioned from 5% to 0.01% CO_2_. Cell samples were collected at 3 h, 6 h, 12 h, and 24 h time points post-transition to the specified conditions for total RNA isolation, followed by mRNA-Seq analysis using Illumina HiSeq2000. Raw reads were processed using TopHat (https://github.com/DaehwanKimLab/tophat, accessed on 10 Febrary 2024) and Cufflinks (https://github.com/cole-trapnell-lab/cufflinks, accessed on 8 Febrary 2024) based on FPKM values (fragments per kilobase of exon model per million mapped fragments).

### 4.4. Plasmid Construction for Gene Knockout via CRISPR

We utilized a previously established procedure for constructing the plasmids used in this study [25]. Briefly, target sequences were selected using the online tool CHOPCHOP ([53]; http://chopchop.cbu.uib.no, accessed on 24 January 2024). Two DNA segments containing the gRNA target sequence and the coding sequence of hammerhead ribozyme (Table S1) were strategically engineered to create a primer dimer through an annealing process. The primer dimer was linked to the pNOC-ARS-CRISPR-v2 vector (hygromycin B) after digestion with a *BspQ*I enzyme. Cas9 and gRNA were driven by a bidirectional *Ribi* promoter. Gene ID of NoLSMT is NO02G01160, which is based on the genome annotation as deposited in NanDeSyn ([54]; http://www.NanDeSyn.org, accessed on 18 January 2024).

### 4.5. Transformation of N. oceanica Cells 

Nuclear transformation of *N. oceanica* was performed for the expression cassette that harbored the endogenous promoters and the Cas9, sgRNA, and HygR genes. Briefly, 100 mL of mid-exponential phase-cultured wild-type *N. oceanica* cells (~4 × 10^7^ cells/mL) were centrifuged at 5000× *g* for 5 min at 4 °C, followed by washing with sorbitol solution and re-suspension in 375 mM cold sorbitol solution to achieve a final concentration of approximately 10^8^ cells/mL. For each transformation reaction, 200 μL of cells that were re-suspended in 375 mM cold sorbitol solution were combined with 2 μg of pNOC-ARS-CRISPR-sgNoLSMT plasmids and 1 μL salmon sperm DNA in an electroporation cuvette before electroporation. Before combination, 1 μL salmon sperm DNA was heated at 95 °C for 1 min for annealing. The cells were then electroporated using an ECM630 BTX electroporator (BTX, Holliston, MA, USA) under specific settings (500 Ω, 50 μF and 2200 V). Post-electroporation, the cells were incubated in customized f/2 liquid medium for 48 h before plating on *hygR*-containing f/2 plates. Resistant colonies were selected after 2–3 weeks and transferred to 24-well culture plates for further liquid culture. Algal cells in the mid-logarithmic growth phase were harvested for DNA extraction in order to confirm successful transformation through PCR amplification. For NoLSMT-CRISPR/Cas9, six lines were validated as positive KO-lines. Among these lines, two knockout types were identified, including ‘GGGGATGGGCTTAGT-GCCACGG’ (NoLSMT-KO-1, single-base-deletion mutagenesis) and ‘GGGGATGGGCTTAGTGG***TTAGGGTTAGGGTT***CCACGG’ (NoLSMT-KO-2, fourteen-base-insertion mutagenesis).

### 4.6. Immunoblot Analysis

Cellular proteins were extracted from 10 to 20 mg of either wild-type or mutant cells utilizing the Pierce^TM^ P-PER plant protein extraction kit (Thermo Scientific, Waltham, MA, USA). Subsequent to protein extraction, Western blot analyses were conducted on the total protein samples obtained from cell lysates after resolving them through sodium dodecyl sulfate-polyacrylamide gel electrophoresis (SDS-PAGE) employing a 12% (*w*/*v*) acrylamide resolving gel by Bio-Rad (Bio-Rad, Hercules, CA, USA). To ensure uniformity, an equal amount of total protein content was loaded in each lane. The separated proteins were subsequently transferred onto a polyvinylidene difluoride (PVDF) membrane (Roche, Nutley, NJ, USA). To prevent the non-specific binding of antibodies, the membrane was blocked with 5% (*w*/*v*) nonfat dried milk in Tween 20 phosphate-buffered saline (TBS; pH 7.4) (Thermo Fisher Scientific, Waltham, MA, USA) for 1 h at room temperature. Following this blocking step, the membranes were incubated overnight at 4 °C with rabbit polyclonal antibodies specific to methylated lysine-N-terminal (ab76118, Abcam, Cambridge, UK) diluted at a ratio of 1:10,000 in phosphate-buffered saline (PBS) (Thermo Fisher Scientific, Waltham, MA, USA) containing 1% (*w*/*v*) nonfat milk. After thorough washing steps, the membranes were then exposed to a secondary antibody, goat anti-rabbit IgG-horseradish peroxidase (HRP) (Thermo Fisher Scientific, Waltham, MA, USA), for the detection process.

### 4.7. Mass Spectrometry-Based Protein Detection

The proteins were initially extracted utilizing an SDS lysis buffer and subsequently subjected to separation on one-dimensional 12.5% (*v*/*v*) polyacrylamide gel through electrophoresis to facilitate the purification and solubilization of the proteins. Following the electrophoresis process, the gel was stained, destained, and digested, enabling the extraction of peptides from the gel pieces in the form of a supernatant, which were then transferred into autosampler vials, specifically 12 × 32 mm^2^ glass screw-neck vials (Waters, Milford, MA, USA). The extracted peptides were then dried using a Speed Vac and stored at room temperature for subsequent analysis. In preparation for the mass spectrometry (MS) analysis of the trypsin-digested proteins, the dried peptides were re-suspended in buffer A (consisting of 0.1% formic acid (FA) in HPLC-grade water (Thermo Scientific, Waltham, MA, USA)) and subjected to sonication for 10 min in an ultrasonic bath (RK-100 H, Heidolph). The LC-ESI-MS/MS analysis, along with protein identification and label-free quantification, were executed according to established procedures and protocols to ensure accurate and reliable results [55].

## 5. Conclusions

In conclusion, our study provides new insights into the function of LSMT in microalgae and its role in carbon fixation. We demonstrated that NoLSMT in *Nannochloropsis* preferentially methylates FBAs rather than Rubisco, and its trimethylation of FBAs may play a regulatory role in the Calvin cycle. The knockout mutants of NoLSMT showed improved biomass accumulation and tolerance to high-light stress, indicating the potential of engineering carbon fixation in microalgae for enhanced biomass production. Further studies are required to fully understand the molecular mechanisms behind, and further optimize this engineering strategy to improve, microalgal carbon fixation.

## Figures and Tables

**Table 1:** Examples of the methods for increasing microalgal biomass.

Method for Increasing Microalgal Biomass	Characteristics	Importance	References
Targeting trimethylation of FBAs through NoLSMT manipulation	- Novel approach to regulate carbon fixation through enzyme modification - Shows promising results in enhancing biomass accumulation and stress tolerance	- Provides new insights into microalgal metabolism and offers potential for significant improvements in biomass production and environmental adaptation	This study
Optimizing growth conditions (light intensity, temperature, nutrient availability)	- Adjusting environmental factors to promote growth and productivity - Relatively simple and widely used method in microalgae cultivation	- Essential for maximizing biomass production and overall efficiency in microalgae-based systems	[39,40]
Genetic engineering of key enzymes (e.g., Rubisco)	- Manipulating enzyme activity to enhance carbon fixation efficiency - Targeted approach to improving specific metabolic pathways	- Offers precise control over metabolic processes, potentially increasing biomass yield	[3,41,42]
Exploiting interactions in co-culture systems	- Leveraging interactions between multiple microorganisms in co-culture systems	- Optimizing the growth environment of microalgae through mutual nutrition and resource exchange, promoting biomass accumulation	[43]
Utilizing wastewater for microalgae cultivation	- Using wastewater as a nutrient source for microalgae growth	- Sustainable approach that reduces environmental impact and production costs	[44]
Application of nanotechnology for nutrient delivery	- Using nanoscale materials to enhance nutrient uptake in microalgae	- Improves nutrient bioavailability and uptake efficiency, leading to enhanced growth and biomass production	[45]
Implementation of biostimulants for growth enhancement	- Utilizing biostimulants to promote microalgal growth and productivity	- Enhances nutrient absorption, stress resistance, and overall performance of microalgae	[46]
Development of high-efficiency bioreactors	- Integration of advanced sensors and automation for real-time monitoring and control of growth parameters - Design features promoting efficient gas exchange and light exposure for enhanced photosynthetic activity	- Enables precise adjustment of environmental factors to optimize microalgae growth and productivity - Facilitates maximum biomass accumulation by providing optimal conditions for microalgae cultivation	[47]
Advantages of NoLSMT Genetic Manipulation			
- Improved biomass accumulation and stress tolerance - Enhanced carbon fixation efficiency - Reduced photoinhibition under high-light conditions - Potential for developing targeted biotechnological approaches to enhance carbon capture in microalgae-based systems			

## Data Availability

Data are contained within the article and supplementary materials.

## Supplementary Materials

The following supporting information can be downloaded at: https://www.mdpi.com/article/10.3390/ijms25073756/s1.
