# Supplementary material for: Knocking Out Chloroplastic Aldolases/Rubisco Lysine Methyltransferase Enhances Biomass Accumulation in Nannochloropsis oceanica under High-Light Stress
Source: Int J Mol Sci. 2024 Mar 28;25(7):3756. doi: 10.3390/ijms25073756 (PMC11012178; doi:10.3390/ijms25073756)
Supplement: Supplementary file 1 [file ijms-25-03756-s001.zip › Table S1. DNA sequences of the primers used in this study.pdf]

Table S1. DNA sequences of the primers used in this study.

Primer for plasmid construction via CRISPR (from 5' to 3')		
**Primer name**	**Forward**	**Reverse**
NoLSMT-KO	CGACACTCTctgatgagtccgtgaggacgaaacgagtaagctcg tcAGAGTGGGGGATGGGCTTAGg	AAAcTCTCACCCCCTACCCGAATCgacgagcttact cgtttcgtcctcacggactcatcagAGAGTG
**Primer for mutants screening** (from 5' to 3')		
**Primer name**	**Forward**	**Reverse**
NoLSMT-Test	TTGCTCACTCCTACACCACAGTC	GGATGTAGACGGCATACTTGGACTTG
